# Anal Canal Gas Gangrene in Aplastic Anaemia: Rare yet Lethal Entity - A Caveat

**DOI:** 10.7759/cureus.1469

**Published:** 2017-07-13

**Authors:** Sundaramurthi Sudharsanan, Chellappa Vijayakumar, Kumar Manish, TP Elamurugan, Ali S Manwar

**Affiliations:** 1 Surgery, Jawaharlal Institute of Postgraduate Medical Education and Research (JIPMER), Puducherry, India.

**Keywords:** gas gangrene, clostridium perfringens, anal canal, debridement, immunocompromised, aplastic anaemia

## Abstract

Gas gangrene is one of the most serious infections of traumatic and surgical wounds. The importance lies in the fact that, if not managed at the right time, the condition is fatal. Though perianal cellulitis and gangrene are commonly reported in immunocompromised patients, we report the case of a patient with gas gangrene involving only the anal canal extending to the rectum, a rare presentation to be reported in literature.

An 18-year-old lady, a patient of aplastic anaemia - immunodeficiency, developed gas gangrene of the anal canal possibly due to faecal contamination of anal fissures. The patient was managed with surgical debridement and intravenous antibiotics.

The clinical manifestations of gas gangrene are due to the liberation of toxins by Clostridium perfringens. The infection spreads rapidly and results in necrosis and devitalisation of tissues, unless intervened surgically.

The clinical manifestations are more rapid and a high index of suspicion is needed for the diagnosis.

## Introduction

Gas gangrene is a rapidly spreading necrotising infection of the skin and soft tissues caused by the bacterium Clostridium perfringens. In most cases, it follows a trauma or a surgery. Spontaneous gas gangrene is rare but can occur in immunocompromised patients [1].

Most physicians and surgeons of today are unfamiliar with the clinical manifestations of gas gangrene, thereby leading to a delay in the diagnosis and appropriate management of these patients. Early identification and aggressive management are vital as the mortality of the disease is very high even in tertiary-care centres.

Gas gangrene involving the ano-genital region is rare, though a few cases of perineal gas gangrene and those involving ischiorectal fossa are reported [2]. We report a case of gas gangrene in an immunocompromised patient involving only the anal canal and extending into the rectum, a rare scenario to be reported in the literature.

## Case presentation

An 18-year-old lady presented with generalised fatigue and excessive bleeding per vaginum for a two-month period. She also had blurring of vision associated with fever and headache for 15 days. The patient complained of pain in the anal region while passing stools for the last one week.

On examination, she was severely pale with an initial pulse rate of 100/min and a blood pressure of 100/70mm Hg. On local examination, she was found to have anal fissures.

On investigating, her peripheral smear revealed pancytopenia with a haemoglobin of 4 g%, total leucocyte count of 490/cu mm and a platelet count of 13000/cu mm. Her bone marrow biopsy was diagnostic of aplastic anaemia. An ophthalmological examination by fundoscopy and a B-scan ultrasonography (USG) was done and she was found to have bilateral vitreous haemorrhage and retinopathy.

She was transfused with multiple units of packed red cells and platelets. She was started on medroxy-progesterone acetate and tranexamic acid for menorrhagia along with iron and folate tablets. She was given cyclosporine for aplastic anaemia. In view of high fever spikes of 103 to 104 degrees F, she was started on empirical ceftazidime and amikacin. Her fever could not be localised and her initial blood and urine cultures were sterile. She had persistent fever and was switched over to meropenam and then to vancomycin.

**Figure 1:**
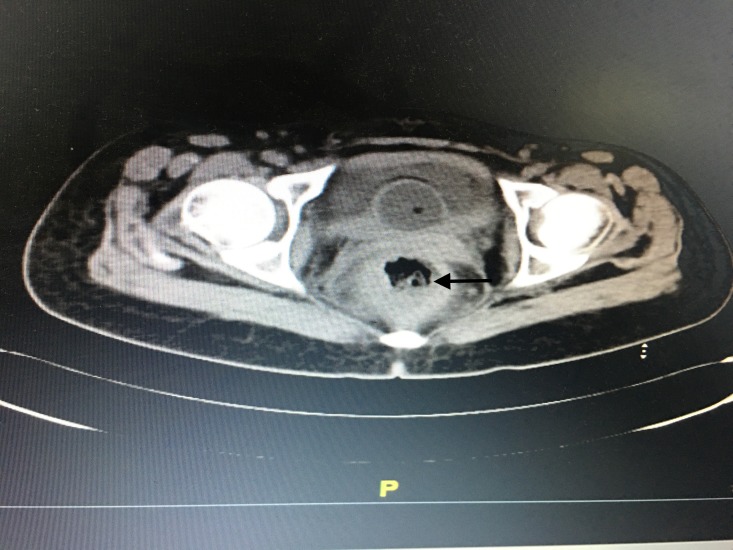
Computed tomography picture showing inflamed anal canal wall with sloughed out mucosa (arrow).

After one week her perianal pain worsened and she passed blood clots per rectum associated with pus discharge. Her anal canal was extremely tender. Computed tomography (CT) scan of her abdomen and pelvis showed features suggestive of perianal abscess with proctitis and sloughed out mucosa in the anal canal (Figure 1).

**Figure 2:**
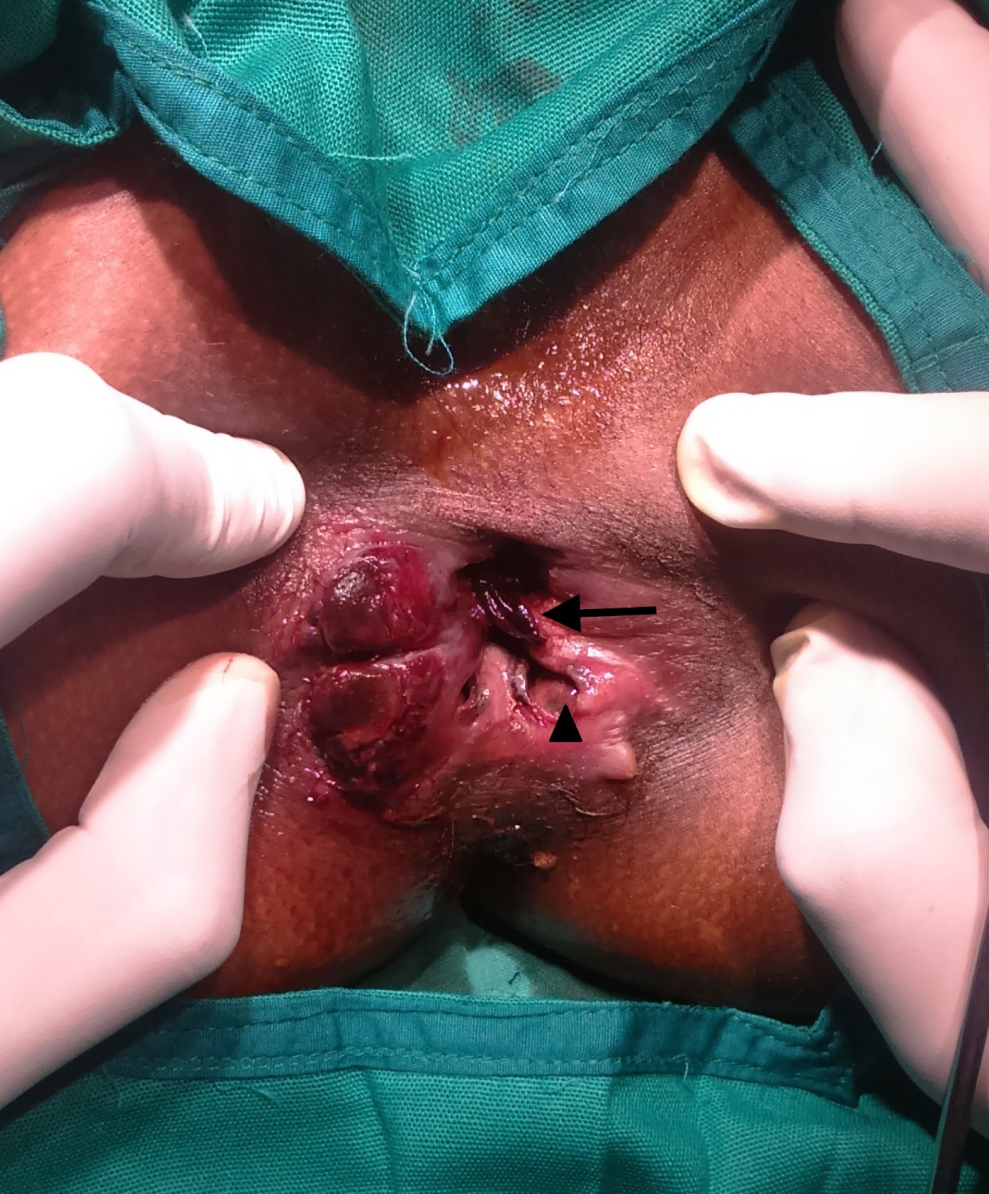
Clinical picture showing devitalised and sloughed out anal mucosa (arrow head) with necrotic tissues and blood clots in the anal canal (arrow).

On examination under anaesthesia, the following findings were noted: the entire anal canal from the anal verge to about 10 cm proximal appeared dark brown in colour with sloughed out mucosa, the sphincters appeared non-viable, the tone was lost, and there was no bleeding on cutting through the tissues. The anal canal was filled with foul-smelling necrotic tissues mixed with blood clots (Figure 2).

**Figure 3:**
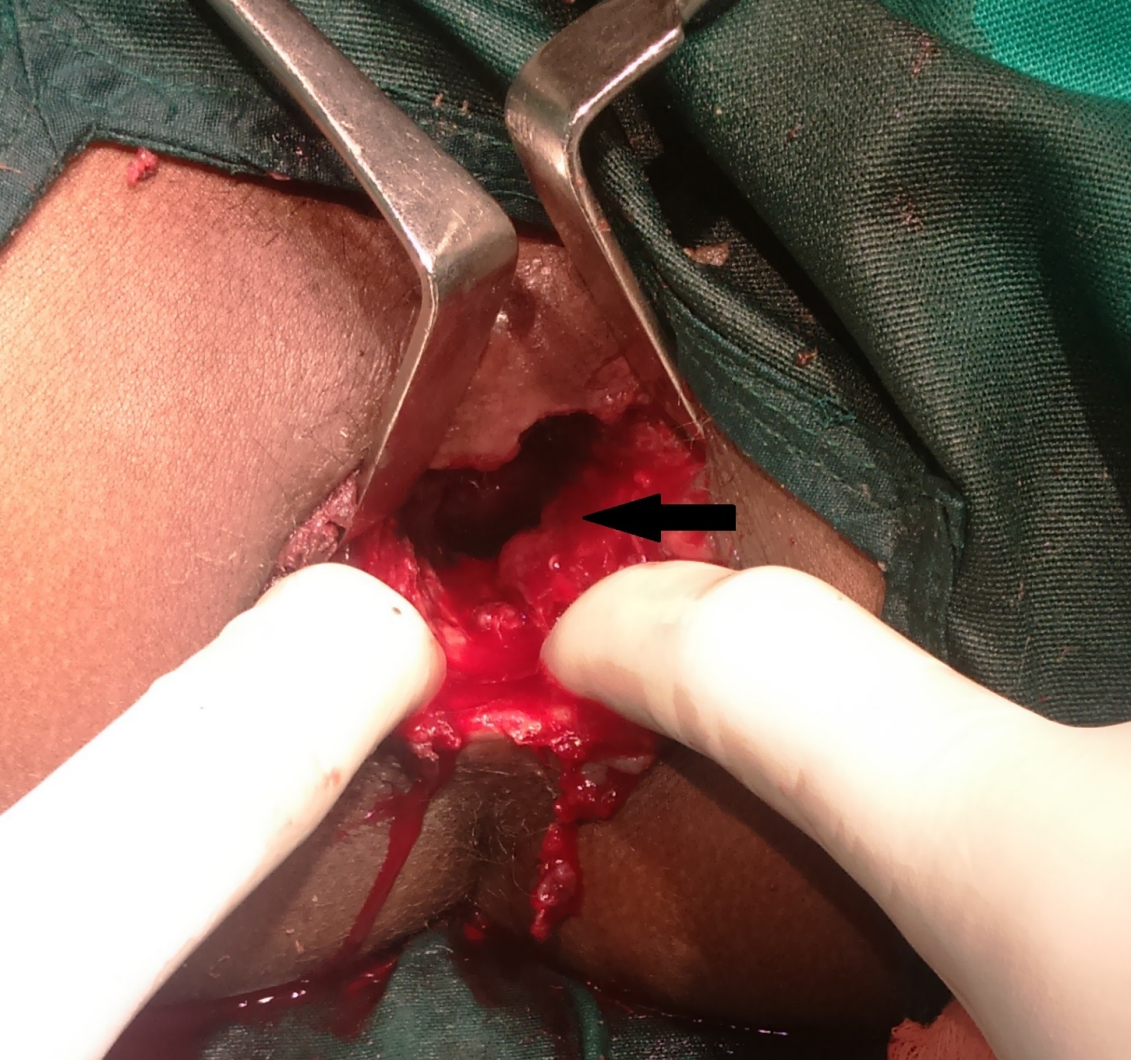
Clinical picture: post-debridement of the necrotic tissues. Healthy bleeding from tissues noted (arrow).

Debridement of the necrotic devitalised tissues was done and the pus collection was drained (Figure 3). A diversion transverse colostomy was made. She was started on piperacillin-tazobactam and clindamycin.

Gram stain of necrotic tissues revealed gram-positive bacilli suggestive of Clostridium along with budding yeast cells. Her subsequent blood cultures grew Clostridium spp and then Escherichia coli (intermediate sensitivity to imipenam) and Acinetobacter baumanii (resistant to all). Her total leucocyte count dropped down to 270/cu mm with an absolute neutrophil count of 30/cu mm. Her general condition worsened. She developed acidosis with the deterioration of vitals. She was resuscitated and was given mechanical ventilation. The patient died of severe sepsis on post-operative day 3.

## Discussion

The causative organism Clostridium perfringens is found in the soil and also as a normal flora in a stool. The organisms gain access to the body through contamination of post-traumatic or surgical wounds and as in this case, through faecal contamination of anal fissures.

The pathology of gas gangrene is due to the exotoxins liberated by the bacteria. The organism liberates a wide variety of toxins, of which the alpha toxin, lecithinase is responsible for most of the clinical manifestations like haemolysis, anaemia, jaundice, and oliguria [3].

Anaerobic environment and the presence of devitalised tissues form a conducive environment for the proliferation of organism and liberation of toxins. The toxin causes damage to the surrounding tissues, especially to the muscles resulting in further access to the tissues by the organism. The toxin hyaluronidase plays a role in the dissolution of the intercellular matrix and helps in the widespread dissemination of the infection. The crepitus is due to the action of proteolytic and saccharolytic enzymes liberating gas in the tissues. The clinical picture is more profound in immunosuppressed individuals, as stool contamination of simple anal fissures may not develop gas gangrene otherwise, in a healthy individual.

The incubation period is less than one day and the infection is rapidly progressive [4]. The clinical picture is marked by an acute onset of severe pain associated with fever, chills, discharge of brownish fluid, and discoloration of skin with surrounding erythema. Local warmth, severe tenderness, and the characteristic crepitus may be present. The infection can lead to fulminant toxemia with resultant hypotension, acute kidney injury, haemolysis, delirium, and even death.

The diagnosis of gas gangrene is clinical and investigations should not delay aggressive surgical treatment and antimicrobial therapy. A gram stain of the tissue exudate or the tissue biopsy will reveal gram-positive rods with few leucocytes. An ultrasound can pick fluid collections and air pockets. In addition, a CT scan can show the extent of the disease process [5].

Early and aggressive management is essential as sepsis develops very rapidly, with a high mortality rate of up to 50% in various studies [6]. Debridement of all the necrotic devitalised tissues and drainage of the collections is needed. The patient might require multiple surgical debridements but the radical removal of all necrotic tissues during the first operation is vital for the survival of the patient. Prompt institution of antimicrobial agents as per the Institute’s protocol is important. The antibiotic spectrum should cover Clostridia, gram-positive and gram-negative organisms as the infection is usually poly-microbial. Hyperbaric oxygen therapy plays a role in preventing extension of the organism to the surrounding uninvolved tissues by increasing the oxygen tension in the surrounding tissues [7]. Gas gangrene serum containing immunoglobulins to neutralise the toxin can be used, but there is no data to support the routine use of immunoglobulins in gas gangrene.

## Conclusions

The management of gas gangrene is challenging and a high index of suspicion is needed, as the presenting complaints may be non-specific. All clinicians must be aware of the clinical presentation of this vital infectious condition and ensure prompt aggressive surgical management. 
